# Chronic Lymphocytic Leukemia Patients Have a Preserved Cytomegalovirus-Specific Antibody Response despite Progressive Hypogammaglobulinemia

**DOI:** 10.1371/journal.pone.0078925

**Published:** 2013-10-23

**Authors:** Katrina Vanura, Franz Rieder, Marie-Theres Kastner, Julia Biebl, Michael Sandhofer, Trang Le, Robert Strassl, Elisabeth Puchhammer-Stöckl, Thomas Perkmann, Christoph F. Steininger, Kostas Stamatopoulos, Wolfgang Graninger, Ulrich Jäger, Christoph Steininger

**Affiliations:** 1 Department of Medicine I, Div. of Hematology and Hemostaseology, Comprehensive Cancer Center (CCC), Medical University of Vienna, Vienna, Austria; 2 Department of Medicine I, Div. of Infectious Diseases and Tropical Medicine, Medical University of Vienna, Vienna, Austria; 3 Department of Virology, Medical University of Vienna, Vienna, Austria; 4 Department of Laboratory Medicine, Medical University of Vienna, Vienna, Austria; 5 Hematology Department and HCT Unit, G. Papanicolaou Hospital, Thessaloniki, Greece; University Hospital San Giovanni Battista di Torino, Italy

## Abstract

Chronic lymphocytic leukemia (CLL) is characterized by progressive hypogammaglobulinemia predisposing affected patients to a variety of infectious diseases but paradoxically not to cytomegalovirus (CMV) disease. Moreover, we found reactivity of a panel of CLL recombinant antibodies (CLL-rAbs) encoded by a germ-line allele with a single CMV protein, pUL32, despite differing antibody binding motifs. To put these findings into perspective, we studied prospectively relative frequency of viremia, kinetics of total and virus-specific IgG over time, and UL32 genetic variation in a cohort of therapy-naive patients (n=200). CMV-DNA was detected in 3% (6/200) of patients. The decay of total IgG was uniform (mean, 0.03; SD, 0.03) and correlated with that of IgG subclasses 1-4 in the paired samples available (n=64; p<0.001). Total CMV-specific IgG kinetics were more variable (mean, 0,02; SD, 0,06) and mean decay values differed significantly from those of total IgG (p=0.034). Boosts of CMV-specific antibody levels were observed in 49% (22/45) of CMV-seropositive patients. In contrast, VZV- and EBV-specific IgG levels decayed in parallel with total IgG levels (p=0.003 and p=0.001, respectively). VZV-specific IgG even became undetectable in 18% (9/50) of patients whereas CMV-specific ones remained detectable in all seropositive patients. The observed CMV-specific IgG kinetics were predicated upon the highly divergent kinetics of IgG specific for individual antigens - glycoprotein B-specific IgG were boosted in 51% and pUL32-specific IgG in 32% of patients. In conclusion, CLL patients have a preserved CMV-specific antibody response despite progressive decay of total IgG and IgG subclasses. CMV-specific IgG levels are frequently boosted in contrast to that of other herpesviruses indicative of a higher rate of CMV reactivation and antigen-presentation. In contrast to the reactivity of multiple different CLL-rAbs with pUL32, boosts of humoral immunity are triggered apparently by other CMV antigens than pUL32, like glycoprotein B.

## Introduction

Patients with chronic lymphocytic leukemia (CLL) are considered immunocompromised based on hypogammaglobulinemia and disturbance of complement activity or neutrophil function that is particularly characteristic for advanced stages of the disease [1]. Accordingly, the 5-year risk for severe infections was 26% overall which increased to 57% when IgG levels were low, and up to 68% for patients with both low IgG levels and disease stage C [2]. In contrast, cytomegalovirus (CMV) disease is not an issue in untreated CLL patients [3,4]. CMV-specific immune response may be preserved even in cases with progressive disease. 

Humoral immunity provides the host with effective instruments for the protection against CMV disease. Total CMV-specific, neutralizing antibodies prevent cell-to-cell spread of the virus, block entry of virions into target cells, and limit viral dissemination in the case of reactivation from latency [5,6]. Accordingly, CMV-seropositive patients with an inborn deficiency in humoral immunity, e.g. chronic variable immunodeficiency, are significantly more prone to CMV disease than immunocompetent individuals [7-9]. In contrast, CLL patients experience a decay of total antibody titers to barely detectable levels but not an increased risk for CMV disease.

CMV-specific antibodies may accordingly follow a different kinetic than the majority of antibodies in CLL patients. Following primary infection, high titers of total CMV-specific antibodies are generated. These antigen-specific antibody titers decrease passively (decay) after clearance of the virus from the circulation but may be boosted upon re-exposure to the specific antigen in the course of reactivation or reinfection with a different CMV genotype [10]. In the absence of immunological boosts, virus-specific antibodies decay exponentially [11,12]. However, knowledge about the kinetic of virus-specific antibodies in association with that of total antibody levels is very limited in healthy individuals and CLL patients. 

CMV expresses more than 20 different immunological targets that differ with respect to immunogenicity and relative frequency of detection by immune sera [13,14]. The antigens most commonly used for vaccine development are envelope proteins (glycoprotein B or H, pentameric complex) because of their high immunogenicity and induction of neutralizing antibody responses. Tegument proteins such as pp65 or pUL32 are hidden within the envelope but are strong inducers of humoral and cellular immune response. pUL32 stands apart from other CMV antigens for several reasons. pUL32 is highly immunogenic in the human host [13,14], is the only polypeptide always reactive in immuno-blotting analyses with immune sera in contrast to all other CMV antigens [15], and is also a strong CD4+ and CD8+ T-cell antigen [13-16]. 

In addition, we found in a previous study that a panel of germline-encoded recombinant antibodies (rAbs) derived from healthy individuals (HI) and leukemic cells from CLL patients reacts with this antigen and none of the other CMV antigens present in whole virus lysates [17]. The antibody binding motifs of these rAbs were highly divergent and still reacted with pUL32 of a single CMV strain. The variability in the signals measured may indicate cross-reactivity among CMV strain-specific antibodies and hence interstrain variability within the antigen coding regions of the UL32 gene [17]. 

Genetic variability within the UL32 gene would possibly have significant immunological consequences similar to other CMV antigens such as glycoprotein B (gB) [18,19]. CMV gB genotypes may differ with respect to their propensity for presentation and recognition by the immune system, modulation of the host´s immune response, virulence, and drug response [20]. Nevertheless, information on the genetic variability of clinical CMV strains within UL32 is very limited despite these wide reaching hypothetical immunological consequences. 

Therefore, the aims of the present study were to evaluate (1) longitudinally the total CMV-specific and the CMV-subclass specific antibody response for two highly immunogenic proteins (pUL32 and gB) in untreated CLL patients at all stages of the disease in comparison to other virus-specific immunoglobulins (2), the frequency of CMV viremia in relation to total CMV-specific antibody titers and (3) the genetic variability of the UL32 gene among clinical CMV strains from CLL patients and the general population. 

## Material and Methods

### Ethics Statement

Written informed consent was obtained from all patients at the time of enrollment. The study protocol was approved by the review board of the Ethics Committee of the Medical University of Vienna in accordance with the Declaration of Helsinki (EK659/2009).

### Patients and clinical samples

EDTA-anticoagulated blood samples were collected from a total of 200 consecutive CLL patients within a prospective cohort study of CLL patients (Collection period: 01.01.2010-31.06.2010). All patients fulfilled the diagnostic and immunophenotypic criteria for CLL [21] and presented for routine evaluation of their disease at the referral center of the Medical University of Vienna. None of the patients had received CLL treatment. Our cohort corresponded to other CLL cohorts reported in the literature [22,23]: median age was 68 years (range 30-92), 54% were men, 46% women. Binet stage A was found in 159 (85.9%), stage B in 17 (9.2%), and stage C in 9 patients (4.9%), no data were available for 15 patients. IGHV gene mutational status was available for 171 patients. From a total of 199 V_H_-genes, 121 (60.8%) were mutated while 78 (39.2%) were unmutated, following the 98% cut-off value for identity to the germline. 

Blood samples were enriched for leukocytes by buffy coat preparation. Before DNA extraction, samples were normalized to a leukocyte concentration of 10^6^ cells/ml. DNA was isolated using QIAamp DNA Blood Mini Kit following the manual (QIAGEN, Valencia, CA, USA). The plasma fraction of these blood samples (n=200) was prepared for testing for total CMV-specific IgG-antibodies. In addition, archived plasma samples were available from 64 of the 200 studied CLL patients. These paired samples were used to determine the change of antibody levels over time. Samples were collected with a mean interval of 4.63 years (SD 1.99 years). Archived bone marrow aspirates were also available from 15 of the 200 CLL patients for evaluation of CMV-DNA positivity. These samples were collected between 11 months before and 6 months after study inclusion (mean -1.1 months; SD 5.6 months).

For comparison of CMV genomic sequences detected in CLL patients with those present in immunocompetent individuals, archived blood samples collected from otherwise healthy patients with primary CMV infection (n=5) and detectable viremia were also included in the investigation. In addition, samples from patients with viremia and multiple myeloma (n=1) or CLL treated with Alemtuzumab (n=2) were included. 

### Serological investigation

CMV-, VZV-, and EBV-specific IgG-antibodies were determined using quantitative ELISAs following the manufacturer’s recommendations (Medac Diagnostika, Hamburg, Germany). The pathogen-specific ELISAs were based on whole virus lysates for CMV and VZV, and on the EBV-specific capsid antigen VCA for EBV, respectively. Levels of total IgG, and IgG_1-4_ subgroup antibodies were determined on a BNII nephelometer (Siemens Healthcare Diagnostic Products, Vienna, Austria) using N Antisera to human IgG, IgG1/2 and N Latex reagent to IgG3/4 (also Siemens Healthcare Diagnostic Products, Vienna, Austria). Reference ranges for IgG were selected according to the recommendation of the test manufacturer (Siemens Healthcare Diagnostics) based on the Consensus reference ranges published by Dati 2001 [24]. Reference values for IgG subclasses (IgG1-4) correspond to the 2.5th to 97.5th percentiles of healthy Central Europe Adults as published by the manufacturer (Siemens Healthcare Diagnostics) in the package insert Edition 2008. 

pUL32- and gB-specific antibodies were determined using an in-house ELISA assay. For this purpose, microtiter plates (Corning Inc., ME, USA) were coated with 25 µl of a sonicated 40µg/ml solution of either mock transfected HEK cells, carrying the plasmid pcDNA3.1, or HEK cells expressing the different CMV antigens. Tris Buffer (50mM Tris, 150mM NaCl, 5%Glycerol) was used for dilution of the cell lysates. Coated plates were stored at 4°C for 12 hours to ensure an optimal binding of the proteins to the wells. 

After washing the plates with 10mM PBS-Tween 20 (0.005%) four times and blocking the wells using StartingBlock Blocking Buffer (Thermo Fisher Scientific, Waltham, MA, USA ), 25µl of human sera diluted at a ratio of 1:500 in blocking buffer were added to each well and the plate was incubated at 4°C for 12 hours. After washing four times with PBS-T20, plates were incubated with anti-human IgG horseradish peroxidase labeled secondary Antibody (Abcam, Cambridge, UK) diluted at a ratio of 1:10.000 for 2 hours at room temperature. After another washing step plates were incubated with 50µl TMB peroxidase substrate (Kirkegaard & Perry Laboratories, MD, USA) for 20 min. The reaction was stopped by adding 50µl 1M *O*-phosphoric acid and the optical density was measured at a wavelength of 450nm.

Patients who received intravenous immunoglobulin preparations before study inclusion (n=2) were excluded from this analysis. Serological diagnosis of CMV-infection status was accomplished in these patients by testing only archived samples collected before immunoglobulin administration for total CMV-specific IgG antibodies.

### Amplification of CMV-specific DNA

All DNA preparations from our cohort (n=200) were tested for the presence of CMV-specific DNA with a nested-PCR assay that amplifies in part the highly conserved CMV UL54 gene (“screening PCR”). This assay was based on a published protocol for a single-step PCR assay [25]. PCR was done using primer pairs CMV12+CMV13 and CMVi12+CMVi13 (Table 1), respectively, using the PCR conditions listed in Table 1. The reaction mixes consisted of 4 µl of sample (primary PCR) or 2 µl PCR reaction (secondary PCR), 2.5µl of 10x PCR buffer, 0.75 µl of MgCl_2_ (50 mM), 2.5 µl of dNTP mix (2 mM each), 0.13 µl iTaq Polymerase (5 U/µl) all obtained from Bio-Rad (Hercules, CA, USA), 0.75 µl of each primer (10 µM), 1.25 µl DMSO (final concentration 5 v/v; Sigma-Aldrich, St. Louis, USA), and ddH_2_O in a final volume of 25 µl. PCR amplicons were visualized on 1% TAE agarose gels. Each PCR experiment included at least one positive control (Bacterial artificial chromosome (BAC)-generated CMV-DNA, kindly provided by D. Spector, University of California, San Diego), and several negative controls. The sensitivity of the UL54-specific PCR was validated using a dilution series of well-defined BAC-generated CMV-DNA in TE buffer indicating an assay sensitivity of 200 copies per ml sample. To validate these data, we spiked DNA preparations from CMV-seronegative patients with the same dilutions of BAC-generated CMV-DNA and attained the same sensitivity. 

**Table 1:** Primers used for amplification and sequencing of UL32.

**Primers**	**Sequence**
**Detection**	
UL54 CMV12-f^1^	GGACCTATTCGTTTTCACACCTAC
UL54 CMV13-r^1^	GTGACAGACACGGCGTATGG
UL54 CMVi12-f^2^	CACACCTACGATCAGACGGA
UL54 CMVi13-r^2^	ATGACCTCACGCAGCCTATC
**Amplification**	
UL32S S-1-r^3^	TTCCTTGATGACGTCGTTTTAGA
UL32S S-2-f^3^,^4^	CTCATGCTTTGGTTGGGATACTA
UL32S S-3-r^4^	TGTTTCTCTAGCCTTCCCTGAAC
UL32D DI-1-f^5^	ATCAAGAAACCGGGAACTAGC
UL32D DII-2-r^5^,^6^	TTCTCGATCTTTTGGAGGATGT
UL32D DI-3-f^6^	CATTTTCAGCGGCATGTTATC
**Sequence analysis**	
UL32S L1-r^7^	TTCTCTAGCCTTCCCTGAACC
UL32S L2-r^7^	TGGACACGGTGTTTTGAGAA
UL32S L3-r^7^	GCTGGCCTTGGTCACCTG
UL32S L4-r^7^	AACGGTTTGTGTCCCTTCC
UL32S L5-r^7^	CACCCTCCAGGTTTTCTTCA
UL32S R1-f^7^	CAACACCGTCGTCCGATTAC
UL32S R2-f^7^	ATCACGGATACCGAGACGAG
UL32S R3-f^7^	CGTGAGAAACAGCAGCTGAA
UL32S R4-f^7^	TGCAAGCTGCTGGTCAAG
UL32D DII-2-r^8^	TTCTCGATCTTTTGGAGGATGT
UL32D L1-r^8^	TAGACGACGGTGGGTAAACG
UL32D R1-f^8^	GTGGCTTTCGACCTATCGTC
UL32D R2-f^8^	CGGTTCAGGGAAGGGTAGAG

f forward primer, r: reverse primer

1 Primer pair for primary UL54 PCR; PCR conditions: 3 min 95°C, 40 cycles 30 sec 95°C, 30 sec 55°C, 30 sec 72°C, final extension 5 min 72°C.

2 Primer pair for secondary UL54 PCR; PCR conditions: 3 min 95°C, 30 cycles 30 sec 95°C, 30 sec 55°C, 30 sec 72°C, final extension 5 min 72°C.

3 Primer pair for primary PCR of UL32 D-segment; PCR conditions: 2 min 94°C, 35 cycles 20 sec 94°C, 30 sec 57°C, 2 min 72°C, final extension 7 min 72°C.

4 Primer pair for semi-nested amplification of UL32 D-segment; PCR conditions: 2 min 94°C, 30 cycles 20 sec 94°C, 30 sec 57°C, 2 min 72°C, final extension 7 min 72°C.

5 Primer pair for primary PCR of UL32 S-segment; PCR conditions: 2 min 94°C, 40 cycles 30 sec 94°C, 30 sec 62°C, 4 min 68°C, final extension 7 min 68°C.

6 Primer pair for semi-nested amplification of UL32 S-segment; PCR conditions: 2 min 94°C, 30 cycles 30 sec 94°C, 30 sec 62°C, 4 min 68°C, final extension 7 min 68°C.

7 Primers for sequencing the UL32 S-segment; reaction conditions: 40 cycles of 20 sec 96°C, 20 sec 50°C and 4 min 60°C.

8 Primers for sequencing the UL32 D-segment; reaction conditions: 40 cycles of 20 sec 96°C, 20 sec 50°C and 4 min 60°C.

In a second step, all DNA preparations that tested positive for CMV-DNA in the UL54-specific PCR assay were tested by two nested PCR assays that amplify the N- and C-terminal half of the UL32 gene of CMV, respectively (“sequencing PCR”). PCR was performed with 0.4 µl of 10 mM dNTP mix (Fermentas Inc., Glen Burnie, Maryland, USA), 1 µl of each primer (10 µM), 0.5 µl of proofreading polymerase (5 U/l), 10 µl 5x PCR reaction buffer without magnesium, 6 µl MgCl_2_ (25 mM) to a final concentration of 3 mM (all part of the Expand High FidelityPLUS PCR System; Roche Applied Science, Mannheim, Germany), 20.6 µl PCR-grade water, 2.5 µl DMSO (5% final concentration), and 8 µl of sample. Semi-nested PCR for amplification of the C-Terminus (termed D-segment) was performed using primer combinations UL32D DII-2+UL32D DI-1 (primary PCR) and UL32D DII-2+UL32D DI-3 (secondary PCR). Amplification of the N-Terminus (S-segment) was done using the primer pairs UL32S S-1+UL32S S-2 (first reaction) and UL32S S-3+UL32S S-2 (second reaction). Primer sequences and reaction conditions are listed in Table 1. 

In case of scarce PCR product, positive bands were cut from 1% TAE agarose gels, purified with Sephaglas Bandprep Kit (GE Healthcare, Piscataway, NJ, USA), followed by ligation into pGEM T-Easy vector and transformation into JM109 cells (both Promega, Madison, WI, USA). Colony PCR of selected positive colonies was performed with standard M13 forward (GTAAAACGACGGCCAG) and reverse (GGAAACAGCTATGACCATG) primers followed by PCR screen with segment specific primers (UL32S S-1, UL32S S-2, and UL32S S-3 for S-segment; UL32D DII-2, UL32D DI-1, and UL32D DI-3 for D-segment) providing sufficient DNA yields for sequencing. 

### Sequencing of the UL32 gene of CMV

PCR products were purified with the GFX PCR Purification Kit (GE Healthcare, Piscataway, NJ, USA) and DNA concentration was measured on a NanoDrop 8000 Spectrophotometer (Fischer Scientific Austria, Vienna, Austria). Primers used in sequencing reactions were UL32S L1, UL32S L2, UL32S L3, UL32S L4, UL32S L5, UL32S R1, UL32S R2, UL32S R3, and UL32S R4 for S-segment; UL32D DII-2, UL32D L1, UL32D R1, and UL32D R2 for D-segment. Primers and reaction conditions are listed in Table 1. Reaction mixes contained 2 µl DMSO (final concentration 10 v/v), 4 µl GenomeLab DTCS Quick Start Kit (Beckman Coulter, Brea, CA, USA), 8 µl primer (1.6 µM), and 60-100 ng of sample. Reactions were precipitated and analyzed with the Beckman CEQ 2000XL DNA analysis system according to the manufacturer´s instructions (Beckman Coulter, Brea, CA, USA).

### Phylogenetic and statistical analysis

Sequences were aligned and edited manually using BioEdit 7.09 (http://www.mbio.ncsu.edu/BioEdit/bioedit.html) and Chromas LITE 2.01 (Technelysium Pty Ltd, http://www.technelysium.com.au). For comparison, the published UL32 sequences of the laboratory strains Toledo, AD169, and Merlin, and the clinical strains 3157, 3301, AF1, HAN13, HAN20, HAN38, JP, TB40/E, U8, U11, and VR1814 were included in the alignment. Phylogenetic distances were determined using the Jones-Taylor-Thornton model for constructing the tree with the maximum likelihood algorithm in MEGA (http://www.megasoftware.net) [26]. Robustness of the nodes was assessed with the Kimura two-parameter model for neighbor-joining algorithms and bootstrap-resampling of 1000 replicates. The probability of variation index was calculated with the software ProbIndex (http://www.cbrg.ethz.ch/) that computes a variation index defined as -log_10_(Probability{position}) for all positions of a multiple alignment. The rate of evolution at each protein site of pUL32 was estimated using maximum-likelihood algorithms in MEGA 5.

Statistical analysis of levels of virus-specific and IgG antibodies was done after transformation of values in log_10_ to symmetries the distribution of values. The decay was calculated as change in log_10_ titer per year. Positive values indicate decay and negative values an increase in antibody levels over time. Statistical analysis of continuous data was carried out using the Spearman rank correlation. Testing for significant differences in the mean ranks between decay of total IgG and decay of virus-specific IgG was done with use of the Wilcoxon signed-rank test. A p<0.05 was considered statistically significant. All statistical analyses were performed using the software SPSS 16.0 (SPSS Inc., Chicago, IL, USA).

## Results

### Productive CMV infection is a rare event in unselected CLL patients

To evaluate untreated CLL patients indirectly for their immuncompetence to limit viral dissemination, we screened all blood samples collected prospectively (n=200) for the presence of CMV-DNA by a CMV-specific PCR assay. None of these patients received specific, anti-CLL therapy at any time before sample collection. CMV-DNA could be detected in 6 of the 200 blood samples (3%), including two samples that were repeatedly tested negative for total CMV-specific IgG antibodies. The six patients positive for CMV-DNA at the time of study inclusion did not have any underlying immunodeficiency other than CLL. Total IgG levels, however, were below the cut-off of normal in 2/6 CMV-DNA positive patients.

To validate the low frequency of CMV-DNA positivity in a second cohort of CLL patients, we also tested retrospectively in an observer-blinded experiment 16 DNA samples from CLL patients that were evaluated in a previous study for the presence of CMV-DNA with the use of a commercially available PCR assay [27]. CMV-DNA was detected correctly in 3/16 samples which corresponded to a sensitivity and specificity of 100%, respectively. In addition, re-analysis of the results obtained from this cohort with inclusion of all CLL subgroups showed comparable CMV-DNA detection rates as in the present cohort (5%, 13 of 253 samples) [27]. 

In addition, we tested archived bone marrow samples which were available from 15 of the present patients because CD34+ precursor cells are one main reservoir of latency for CMV. All of these bone marrow samples tested negative for CMV-DNA.

### CMV-specific antibody titers are boosted frequently despite decay of total IgG

**Figure 1:**
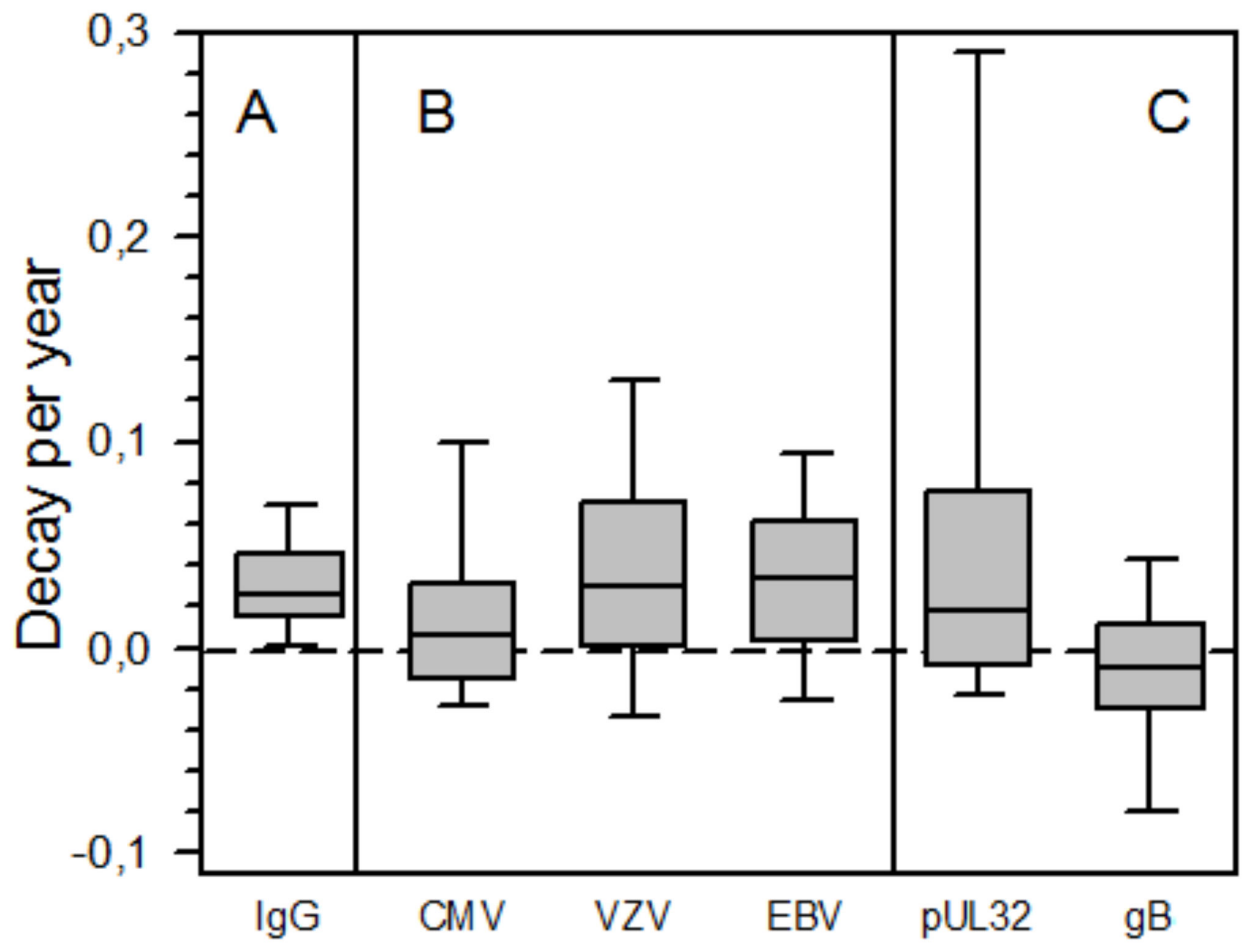
Decay per year of antibody levels in paired samples from CLL patients (n=64). Changes of levels of total IgG and of VZV- and EBV-specific IgG were determined in paired samples of 64 CLL patients. From the same patient cohort, paired samples of the 45 CMV-positive patients were screened for total CMV-specific IgG and IgG specific for the large CMV phosphoprotein pUL32, and for CMV glycoprotein B. Decay was calculated as change in log_10_ titer per year, positive and negative values indicating decrease and increase in antibody levels over time, respectively. A) Changes of levels of total IgG; B) Changes of CMV-, VZV-, and EBV-specific IgG; C) Changes of pUL32- and gB-specific IgG. Horizontal line indicates constant Ig levels. P-values were 0.034, 0.612, and 0.498 for total IgG vs. CMV-specific, VZV-specific, and EBV-specific IgG. P-values were 0.655 and <0.001 for total IgG vs. pUL32-specific and gB-specific IgG, respectively [Wilcoxon Signed Ranks Test].

In contrast to the low detection rate of CMV-DNA in blood samples from the CLL patients, total CMV-specific IgG antibodies were found in 71.5% (143 of 200) of the samples indicative for a high proportion of patients latently infected with CMV. To determine the change in total CMV-specific antibody levels over time, we quantified in parallel total CMV-specific IgG in archived samples available from the presently studied patients (n=64; mean interval between collection of samples 4.63 years; SD 1.99 years). Total CMV-specific IgG were detected in 45 (70%) of the archived samples. Analysis of the change in total CMV-specific IgG levels over time showed an increase in total CMV-specific IgG levels in 22 of the 45 seropositive patients (48.9%) as indicated by negative decay values (Figure 1). Total CMV-specific IgG levels did not fall below the cut-off for positivity in any of the CMV-seropositive patients during the periods of observation and none of the patients seroconverted from negative to positive CMV-IgG values during the period of observation (Table 2). In contrast, all other virus-specific Ab titers evaluated showed an even higher variability in Ab kinetics but almost uniformly a decay of Ab levels with few boosts in titers (Figure 1). Seroconversion from positive to negative IgG values was noted in several patients for EBV- and, most frequently, for VZV-specific IgG (Table 2). Seroconversion from negative to positive IgG values was observed only in one patient for VZV-specific antibodies indicative for a primary infection. 

**Table 2:** Pathogen-specific immunglobulin serostatus and serokinetics in paired samples from 64 CLL patients.

	**CMV**	**VZV**	**EBV**
No. seropositive (%)	45/64 (70.3)	50/58 (86.2)	56/58 (96.6)
No. seroconversion from positive to negative (%)	0/45 (0)	9/50 (18.0)	2/56 (3.6)
No. seroconversion from negative to positive	0	1	0
Decay of virus-specific Ab per year; mean (SD)^1^	0.017 (0.06)	0.029 (0.12)	0.033 (0.05)

1 Decay of total IgG per year; mean (SD): 0.03 (0.03).

Seropositivity was determined in the first sample collected from the respective patient, conversion of serostatus was determined by comparing qualitative test results for paired samples per patient. Mean interval between the collection of the two samples was 4.63 years (SD 1.99 years).

CMV-subclass pUL32-specific IgG levels did not follow the same kinetics as total CMV-specific IgG (mean 0.062; SD, 0.19) (Figure 1). Overall, CMV-subclass pUL32-specific IgG declined in a majority of cases, boosts of antibody titers were observed in 32% (13/41) of patients. In contrast, levels of antibodies specific for CMV-subclass glycoprotein B did not decline but increased in 51% (19/37) of patients (mean -0.0095; SD, 0.045)(Figure 1) indicating regular boosts of this class of antibodies. 

In order to put the observed variability of virus-specific IgG levels in context with the known decrease in total IgG levels, we analyzed also the kinetics of total IgG and IgG subclass levels in the same set of paired samples (n=64). Levels of total IgG were above the lower limit of normal in 37 of 64 (58%) of the archived samples and this rate fell to 34% during the observation period. The decrease in immunoglobulin levels per year was comparable for all of the samples tested and, in contrast to levels of total CMV-specific IgG, decreased with very limited variability (Figure 1, Table 3). The mean decay per year was significantly different between total IgG and total CMV-specific IgG (p=0.034), total IgG and CMV-subclass gB-specific IgG (p<0.001), but not between total IgG and VZV-, EBV-, and CMV-subclass pUL32-specific IgG (Figure 1; p-values being 0.612, 0.498, and 0.655, respectively; [Wilcoxon Signed Ranks Test]).

**Table 3:** Immunoglobulin serum levels and decay of antibodies per year in paired samples from 64 CLL patients.

		**IgG (mg/dl)**	**IgG1 (mg/dl)**	**IgG2 (mg/dl)**	**IgG3 (mg/dl)**	**IgG4 (mg/dl)**
	Normal ranges	700-1600	405-1011	169-786	11-85	3-201
1° Sample	Median	781.0	519.0	218.0	30.3	18.1
	Range	211.0-1700.0	155.0-935.0	45.3-739.0	3.2-101.0	1.8-98.6
2° Sample	Median	583.0	430.0	155.5	22.9	8.3
	Range	201.0-1540.0	119.0-1080.0	22.2-480.0	1.1-69.8	0.8-74.6
Decay of Ab per year	Mean (SD)	0.03 (0.03)	0.02 (0.03)	0.04 (0.03)	0.04 (0.04)	0.06 (0.04)

Median values and ranges of total IgG and IgG subclass levels in the subgroup of CLL patients for which paired samples were available (64/200). Mean interval between the collection of the archived sample (1° Sample) and the sample collected at study inclusion (2° Sample) was 4.63 years (SD 1.99 years). Reference ranges for IgG were selected according to the recommendation of the test manufacturer (Siemens Healthcare Diagnostics) based on the Consensus reference ranges published by Dati 2001 [24]. Reference values for IgG subclasses (IgG1-4) correspond to the 2.5th to 97.5th percentiles of healthy Central Europe Adults as published by the manufacturer (Siemens Healthcare Diagnostics) in the package insert Edition 2008. Antibody decay was calculated as change in log_10_ titer per year, values represent the mean (standard deviation) of the 64 sample pairs for each antibody subclass.

**Figure 2:**
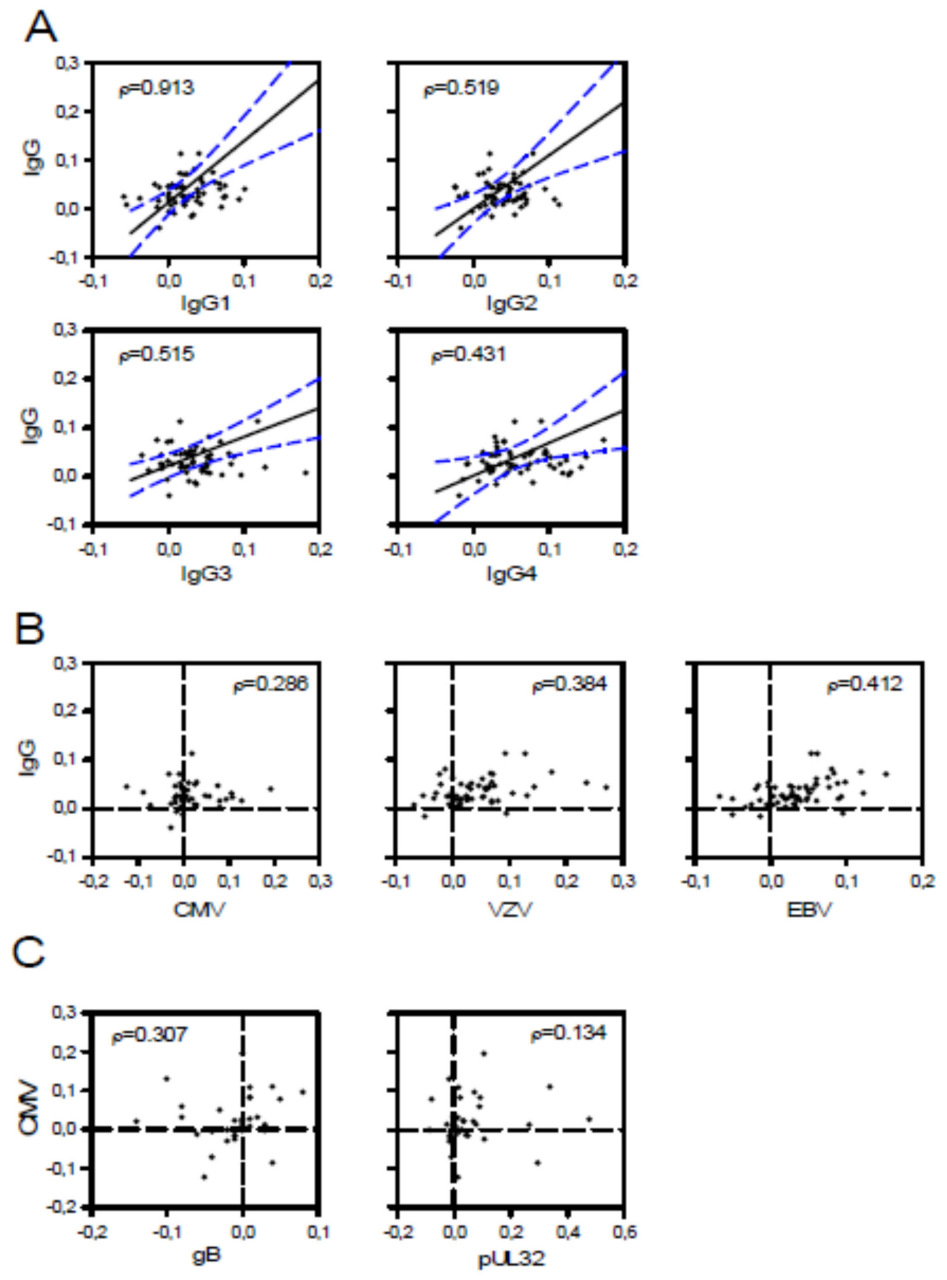
Kinetics of antibody levels in paired samples from CLL patients (n=64). The decay constant λ was calculated as mean change in log_10_ titer per year. Positive values of λ indicate decay and negative values an increase in antibody levels over time. Statistical analysis was done using Spearman´s rho correlation coefficient which is shown for each comparison. (A) Decay per year of IgG subgroups plotted against total IgG decay. Lines indicate linear correlation and 95% confidence intervals, respectively. Decay of total IgG strongly correlated with that of IgG subgroups, p-values being <0.001 for all comparisons tested (not shown). (B) Decay constants of total IgG plotted against CMV-specific, VZV-specific, and EBV-specific IgG. Correlation between decay constants were statistically significant for total IgG and VZV- and EBV-specific IgG (p-values 0.003 and 0.001, respectively), while correlation between total IgG and CMV- specific IgG was not significant (p=0.087). (C) Decay constants of CMV-specific IgG plotted against CMV-subclass gB-specific and CMV-subclass pUL32-specific IgG. Correlations of decay constants were unspecific for both comparisons (p-values 0.068 and 0.405, respectively).

Similarly, levels of IgG subclasses 1 through 4 followed the same kinetics as that measured for total IgG (Table 3). Comparison of the decay of total IgG levels over time with that of levels of IgG subclasses showed a linear correlation in all cases, respectively (p<0.001 [Spearman Correlation Coefficient]; Figure 2A). This indicates that the development of hypogammaglobulinemia affects all subclasses of IgG equally. VZV- and EBV-specific antibody kinetics but not those of CMV-specific antibodies correlated with total IgG kinetics (Figure 2B; p=0.003, p=0.001, and p=0.087, respectively) indicative for rare boosts of humoral immunity against VZV and EBV in the cohort studied. In contrast to the correlation between the decay of total IgG and that of IgG subclasses, total CMV-specific IgG levels did not correlate with CMV-subclasses pUL32-specific and gB-specific IgG (Figure 2C; p=0.405 and 0.068, respectively). 

### UL32 sequences are highly homologous among clinical CMV strains

**Figure 3:**
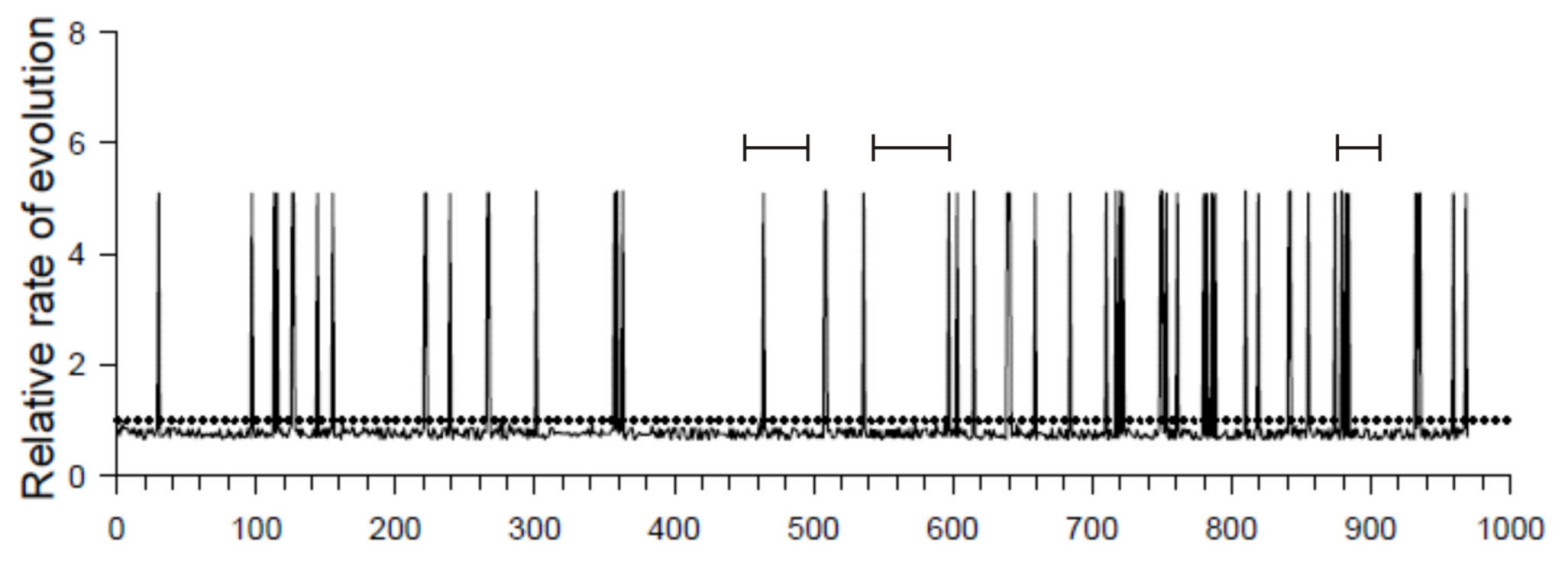
Relative rate of evolution of the UL32 gene based on amino acid-sequence. The mean evolutionary rate per amino acid site within the UL32 gene was calculated to identify genomic regions with a more than average genomic variation. Relative evolutionary rates are shown for each site next to the site number. These rates are scaled such that the average evolutionary rate across all sites is 1 (dotted line). This means that sites showing a rate < 1 are evolving slower than average and those with a rate > 1 are evolving faster than average. These relative rates were estimated with the use of MEGA under the Jones-Taylor-Thornton model (+G) (21). Known immunogenic epitopes are indicated by horizontal bars above the graph [28-30].

**Figure 4:**
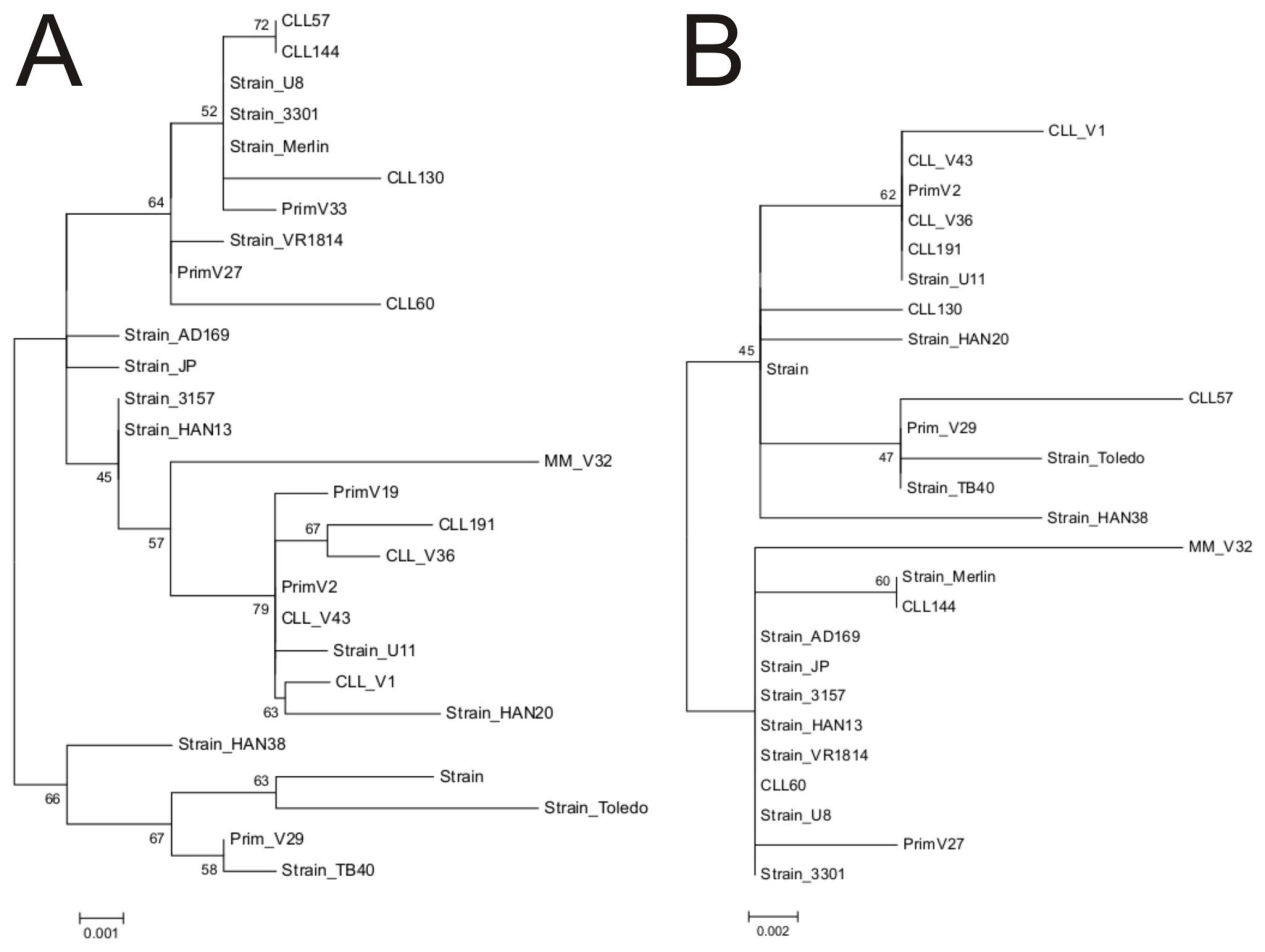
Phylogenetic analysis of the CMV UL32 gene. (A) Analysis of the N-terminal aa50-784 of the UL32 gene from CMV strains detected in our study (CLL, n=7; multiple myeloma, n=1; immunocompetent patients with primary CMV infections, n=5). (B) Analysis of the C-terminal aa493-1037 of the UL32 gene from the same CMV strains (CLL, n=7; multiple myeloma, n=1; immunocompetent patients with primary CMV infections, n=3). Sequences were compared to the published sequences of 3 laboratory adapted CMV strains and 11 previously described clinical isolates (see methods section) using the Jones-Taylor-Thornton model for constructing the tree with the maximum likelihood algorithm in MEGA. Robustness of the nodes was assessed with the Kimura two-parameter model for neighbor-joining algorithms and bootstrap-resampling. Bootstrap values (% after 1000 iterations) are shown for major branches.

To investigate the genetic variability within the UL32 gene, we amplified, sequenced, and analyzed the UL32 gene from the CMV-DNA positive samples (5/6). To validate findings, we also evaluated the UL32-sequences from CMV strains detected in samples from other patients with CLL (n=2) or multiple myeloma (n=1), and from immunocompetent patients with primary CMV infection (n=5). Comparison of these sequences revealed that more than 99% of the entire UL32 gene is homologous among the clinical CMV strains studied and the reference strains of CMV published previously (AD169, Merlin, Toledo, TB40/E). The limited genetic variability observed was located outside the reported regions encoding immunogenic epitopes and distributed stochastically over the entire UL32 gene as demonstrated by the analysis of the relative rate of evolution per amino acid position (Figure 3). Alignment of the genomic sequences with the use of AD169 as reference strain showed that the variability was based on point mutations resulting in the substitution of a single amino acid (Figure S1). Insertion of an amino acid in comparison to the reference strain was observed only at two positions - insertion of a Val at position aa846 and insertion of one or two Asp at position aa383. Phylogenetic analysis of the available UL32-sequences with the use of maximum-likelihood and neighbour-joining algorithms showed that differentiation into several UL32-genotypes was not possible in view of the low genetic variability - phylogenetic trees generated with the use of different algorithms had the same overall topology (Figure 4).

## Discussion

In the present study, we demonstrate that the total CMV-specific antibody response remains preserved in therapy-naïve CLL patients despite an overall decay of total IgG. Total CMV-specific IgG levels even increased in half of the CLL patients studied indicative of presentation of CMV antigens and consecutive boost of total CMV-specific IgG levels. Presentation of CMV antigens was not associated with prolonged periods of viremia or CMV disease. Accordingly, the quantitative and qualitative CMV-specific immune response appeared to be preserved in the presently studied patients. In contrast, other virus-specific IgG titers mostly followed the same kinetic as total IgG, even those specific for the closely related EBV, indicative of a more frequent CMV-specific challenge of the immune system compared to the other viral pathogens studied. These observations underline also the supreme importance of containing and neutralizing CMV which bring about the dedication of significant resources of the host’s immune system against CMV infection. 

Detection of CMV-DNA in serum samples from immunocompetent individuals is an extremely rare event despite the use of highly sensitive PCR assays [10]. We screened leukocyte preparations for the presence of CMV-DNA which carry the highest CMV-DNA copy numbers of all blood fractions during CMV latency [31]. Still, CMV-DNA was detected only in 3% of samples tested. The detection rate of CMV-DNA in serum samples observed in our cohort is almost identical to the rate observed in a previously studied CLL cohort from a different geographic region [27]. CMV disease was observed in none of the CLL patients of the two cohorts. These observations provide indirect evidence for a preservation of the quality of the CMV-specific immune response which stands in contrast to the loss of immunoglobulins with progression of the disease. 

The evaluation of the quantity of the antibody response over a mean period of 5 years revealed that the kinetics of total immunoglobulins differ clearly from that of the total CMV-specific antibody response. Passive loss of IgG and IgG subclasses follows a logarithmic function [11,12] – in our study with the decay constant λ of 1.07. The calculation of the biological half life of total IgG in CLL patients with the use of this constant may allow the prediction of the time when IgG levels are critically low and risk for opportunistic infections is high. In contrast, longitudinal changes in total CMV-specific IgG were variable. Levels of total CMV-specific IgG increased in about half of the CLL patients indicative of boosts of CMV-specific immunity during the observation periods. CMV antigens may have been presented and CMV-specific immunity stimulated consistent with the short and extremely infrequent episodes of CMV viremia occurring in immunocompetent, healthy individuals [10]. 

The pUL32-specific Ab titers surprisingly followed different kinetics compared to total CMV- and gB-specific Ab titers. Total CMV-specific and gB-specific Ab titers were clearly more frequently boosted than those of pUL32-specific Abs. It may only be speculated on the potential reasons for this observation. pUL32 is essential for viral replication [32-34] and highly abundant in the mature virion [35,36]. During latency, viral gene expression is limited to early genes which does not include gB or pUL32 [37]. Hence productive replication of CMV is always associated with generation of pUL32 and lack of this antigen may not explain discrepant Ab kinetics [36]. Alternatively, processing, presentation, and recognition of pUL32 may be different from other antigens. In contrast to envelope proteins such as gB, pUL32 does not induce neutralizing antibodies following primary infection [13]. gB-specific antibodies limit viral dissemination within the human host and periods of viremia - hypothetically faster than UL32 may be processed and presented to immune cells to boost pUL32-specific immunity [10]. Thus pUL32 is highly immunogenic but appears to be a subdominant target for the boost of healthy B-cells, although further studies are warranted to clarify this matter.

All other virus-specific Ab levels evaluated decreased overall in parallel with the decay of total IgG titers. A considerable number of CLL patients even seroconverted from detectable to undetectable virus-specific antibodies during the period of observation. These patients may have been classified erroneously as uninfected despite a latent VZV or EBV infection. Accordingly, antibody-mediated immunity remains responsive to the frequent presentation of CMV Ags and more extensively than to presentation of other viral Ags despite progressive hypogammaglobulinemia in CLL patients. 

The same may pertain to virus-specific cellular immunity. In CLL patients, the total number of T-cells is increased, CD4+:CD8+ T-cell ratio reversed, and T-cells exhibit a number of functional defects [38,39]. Still, CMV-specific T-cell responses are preserved, relative frequency of both CD4+ and CD8+ CMV-specific T-cells increased, and CMV antigens remain a target for cytotoxic T-cells [38,40,41]. Immune senescence in healthy individuals is also characterized by an expansion of both CMV-specific CD4 and CD8 T-cells and an inversion of CD4+:CD8+ ratio [42-44]. CMV viremia is rare in both cohorts and only additional hits to the immune system lead to the high frequency of symptomatic CMV infections observed for instance in CLL patients treated with the humanized monoclonal antibody Alemtuzumab that targets CD52+ leukocytes (CMV-seroprevalence has not been assessed to date in this patient group) [3,45-47]. Preservation of a functional humoral and cellular immune response to CMV infection appears to be of major evolutionary significance for the human host in health and disease.

We observed in a recent study binding activity of multiple CLL rAbs and VH1-69-encoded rAbs with different antigen-binding motifs with pUL32 [17]. Still, we found that the genomic variability within the sequences derived from the different clinical CMV strains was below 1%. The low genomic diversity of UL32 observed is in concordance with that found in the few previously studied clinical CMV strains and was similar between CMV strains detected in CLL patients and immunocompetent individuals. These observations support our previous notions that pUL32 might be a B-cell superantigen [17]. Superantigens are microbial proteins which i) are oligo- and multivalent, ii) show broad reactivity with a large fraction of the B-cell pool and iii) induce activation and proliferation of B-cells [48]. B-cell superantigens target particularly natural, germline-encoded antibodies (IgM-like Abs) which serve as first defense against common pathogens [49]. Superantigens bind to conserved regions outside of the conventional Ag binding site involving regions common to structurally related sets of V genes [48,49]. In concordance, we found that pUL32 derived from a single CMV strain reacted with a panel of different CLL rAbs and IGHV1-69 rAbs from healthy donors but not detectably with sera from CMV seronegative individuals. Thus, considering the regular boosts of CMV-specific Abs, one would also expect persistent and continuous stimulation of pUL32-specific antibodies. The rare boosts of pUL32-specific Abs observed in this study, despite the strong immunogenicity of this viral protein, contrasts markedly with this assumption but would further support our hypothesis that CMV virions are rapidly eliminated from the circulation after maturation of the CMV-specific immunity, more rapidly than UL32-specific Abs are boosted [10]. 

In conclusion, CLL patients have a preserved CMV-specific antibody response despite progressive decay of total IgG and IgG subclasses. The exceedingly low rate of CMV infection and disease may be explained in part accordingly considering also the significance of cellular CMV-specific immune response. The control of CMV and frequent reactivations from latency by dedicating major resources of the immune system to this task seems critical for the human host from an evolutionary point of view. 

## Supporting Information

Figure S1 **Alignment of the UL32 amino acid (aa) sequences.**
(DOCX)
