# Supplementary material for: Chronic Lymphocytic Leukemia Patients Have a Preserved Cytomegalovirus-Specific Antibody Response despite Progressive Hypogammaglobulinemia
Source: PLoS One. 2013 Oct 23;8(10):e78925. doi: 10.1371/journal.pone.0078925 (PMC3806856; doi:10.1371/journal.pone.0078925)

**Figure S1:** Alignment of the UL32 amino acid (aa) sequences compared in this study (n=27), the clinical strain AD169 was used as reference strain (first row). Dots represent the same aa as in the reference strain, dashes represent gaps, aa changes are denoted using the standard one letter code.


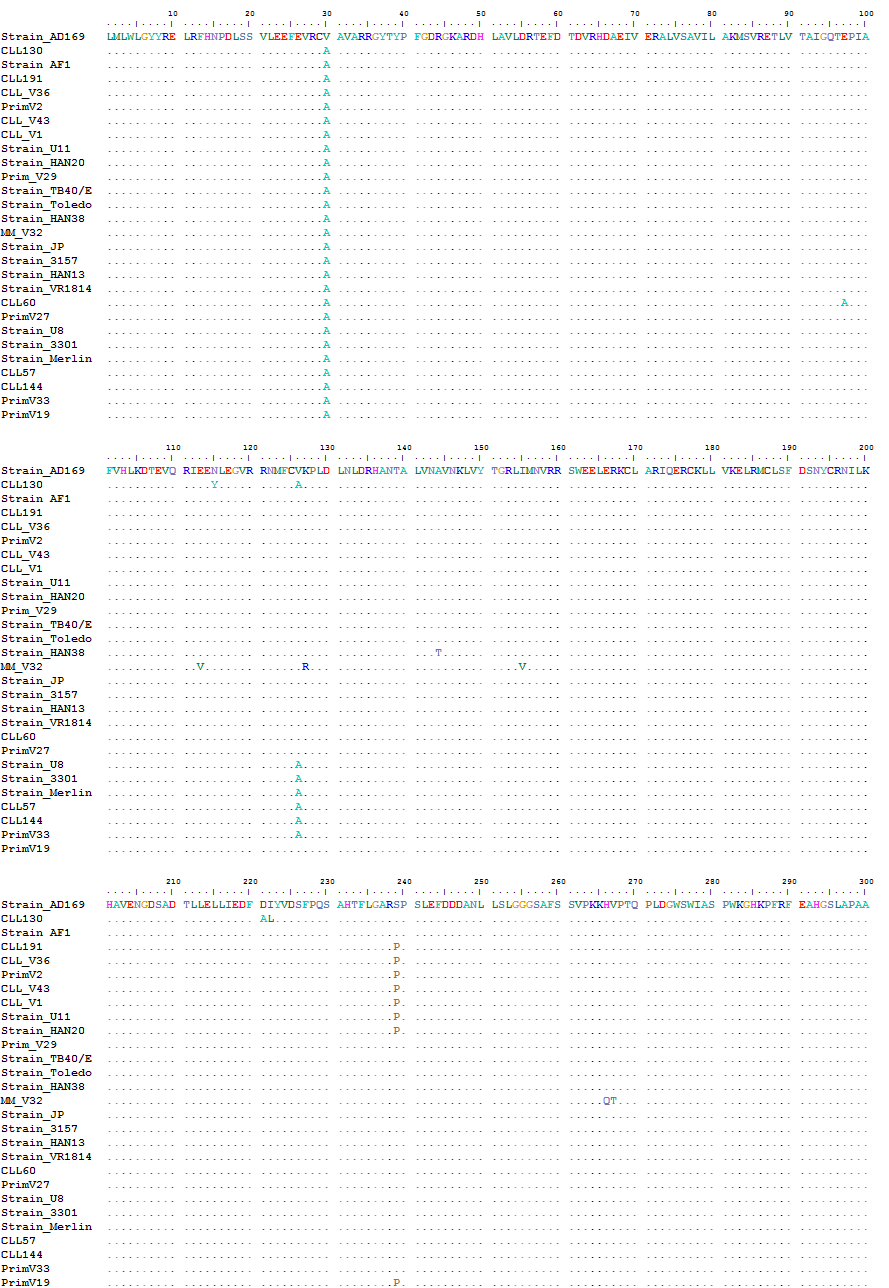

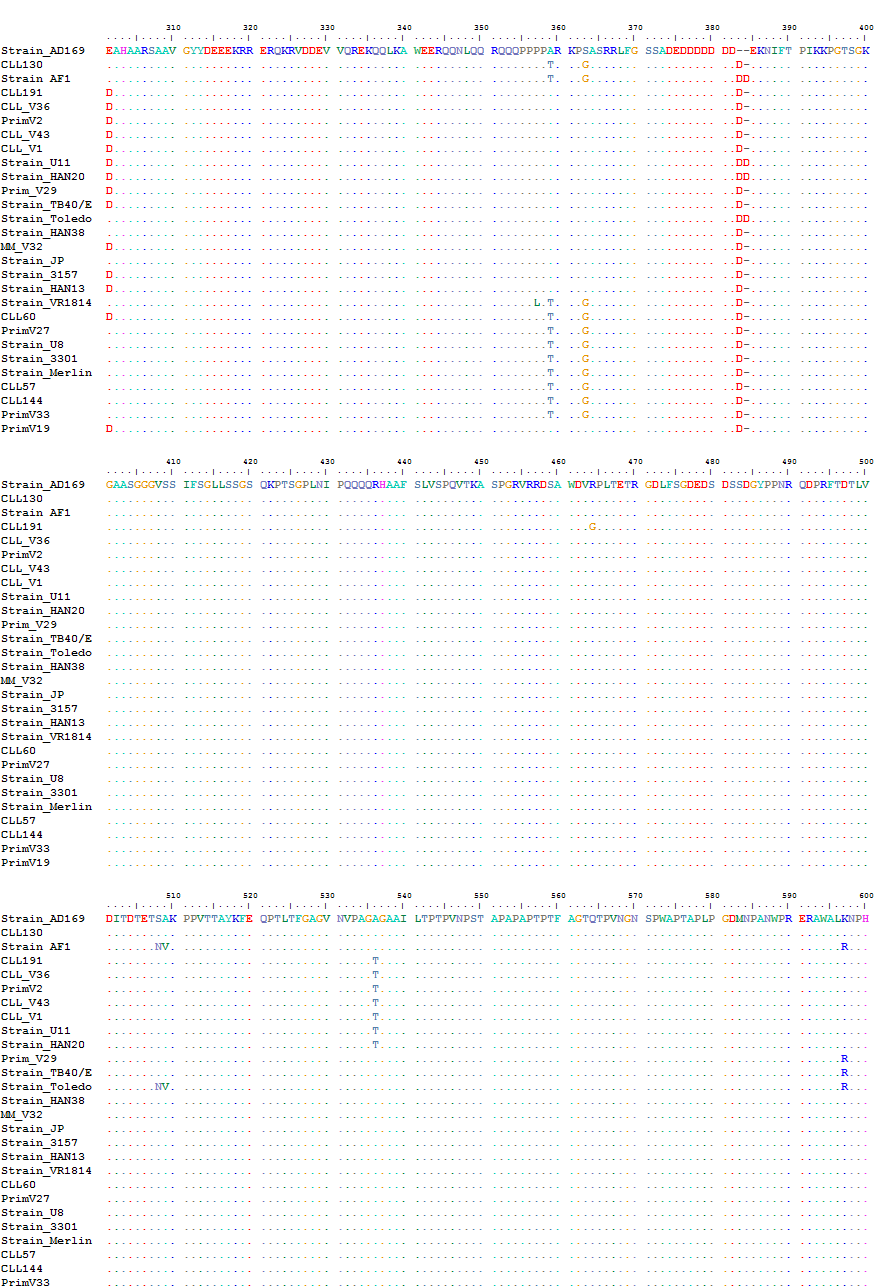

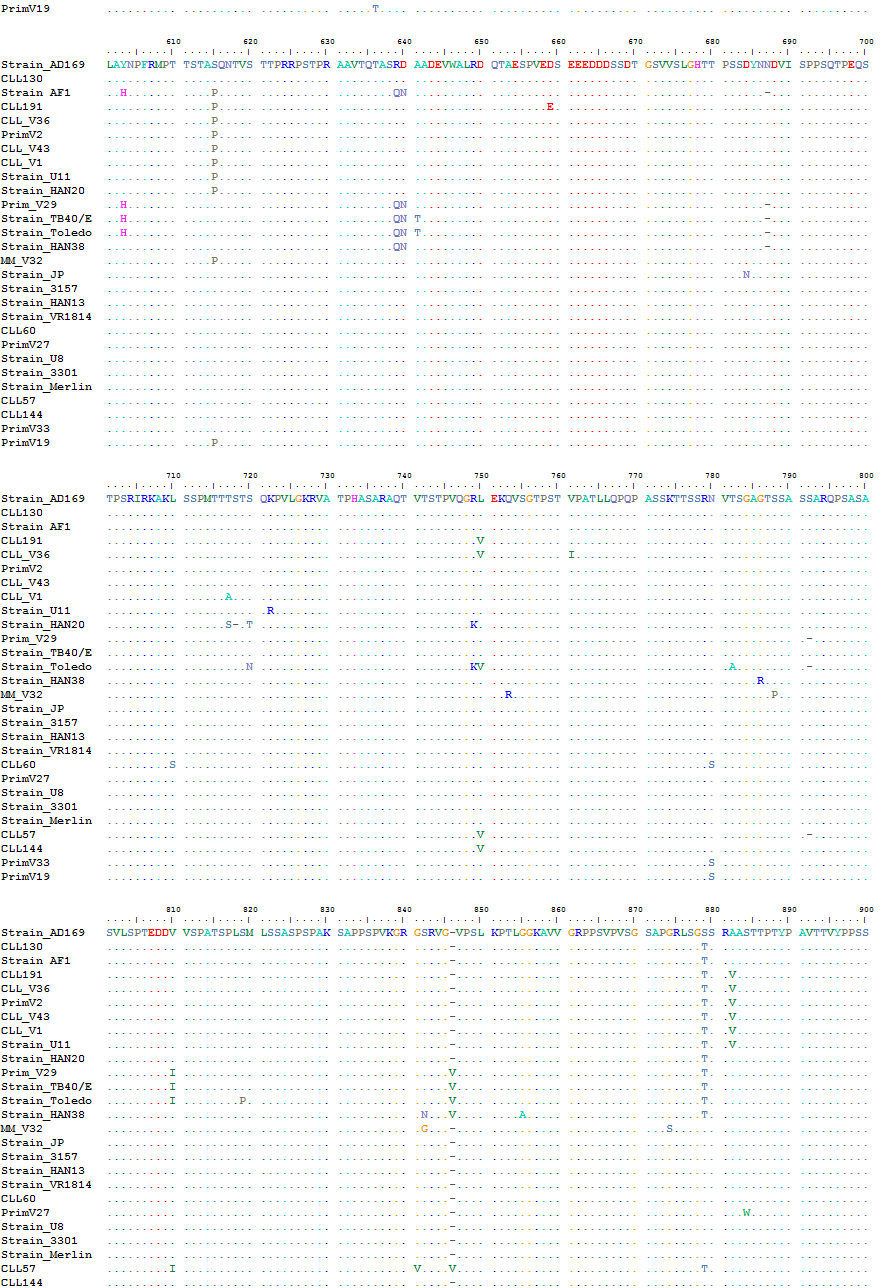

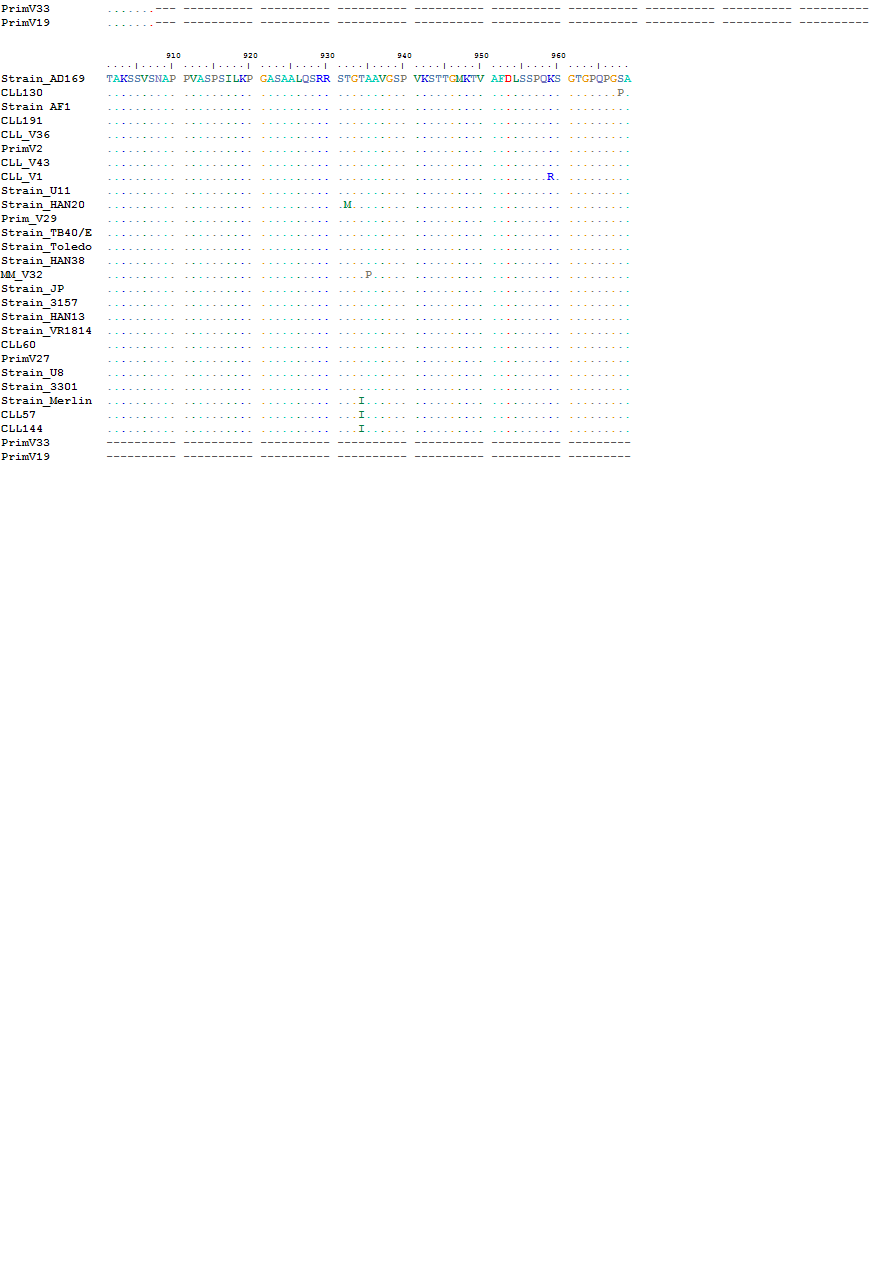

Supplement: Figure S1 — Alignment of the UL32 amino acid (aa) sequences. (DOCX) [file pone.0078925.s001.docx]
